# Hyperspectral imaging with deep learning for quantification of tissue hemoglobin, melanin, and scattering

**DOI:** 10.1117/1.JBO.29.9.093507

**Published:** 2024-09-06

**Authors:** Thomas T. Livecchi, Steven L. Jacques, Hrebesh M. Subhash, Mark C. Pierce

**Affiliations:** a Rutgers, The State University of New Jersey, Department of Biomedical Engineering, Piscataway, New Jersey, United States; b Colgate-Palmolive Company, Global Technology and Design Center, Piscataway, New Jersey, United States; c University of Washington, Department of Bioengineering, Seattle, Washington, United States

**Keywords:** hyperspectral imaging, deep learning, blood volume, oxygen saturation, melanin, skin, gingiva

## Abstract

**Significance:**

Hyperspectral cameras capture spectral information at each pixel in an image. Acquired spectra can be analyzed to estimate quantities of absorbing and scattering components, but the use of traditional fitting algorithms over megapixel images can be computationally intensive. Deep learning algorithms can be trained to rapidly analyze spectral data and can potentially process hyperspectral camera data in real time.

**Aim:**

A hyperspectral camera was used to capture 1216×1936  pixel wide-field reflectance images of *in vivo* human tissue at 205 wavelength bands from 420 to 830 nm.

**Approach:**

The optical properties of oxyhemoglobin, deoxyhemoglobin, melanin, and scattering were used with multi-layer Monte Carlo models to generate simulated diffuse reflectance spectra for 24,000 random combinations of physiologically relevant tissue components. These spectra were then used to train an artificial neural network (ANN) to predict tissue component concentrations from an input reflectance spectrum.

**Results:**

The ANN achieved low root mean square errors in a test set of 6000 independent simulated diffuse reflectance spectra while calculating concentration values more than 4000× faster than a conventional iterative least squares approach.

**Conclusions:**

*In vivo* finger occlusion and gingival abrasion studies demonstrate the ability of this approach to rapidly generate high-resolution images of tissue component concentrations from a hyperspectral dataset acquired from human subjects.

## 1 Introduction

Biological tissues contain a complex mixture of components, including blood, water, fat, and collagen. The quantification of these components has relevance in physiology and diagnosis of disease. For example, melanoma diagnosis can benefit from accurate melanin quantification,^1^ whereas peripheral arterial disease diagnosis can be improved through precise blood volume and oxygen saturation measurements.^2^ Traditional methods such as blood sampling or tissue biopsy are invasive and provide information from a small biological sample at a single point in time. Non-invasive probe-based optical spectroscopy can quantify tissue blood content, oxygen saturation, water, lipid, and melanin content but lacks spatial information.^3^^–^^10^ Imaging approaches including planar multi-spectral reflectance imaging,^11^^,^^12^ spatial frequency domain imaging,^13^^–^^17^ and diffuse optical spectroscopic imaging^18^ suffer from either limited spatial or spectral resolution.

Spectral imaging integrates conventional imaging and spectroscopy to attain both high-resolution spatial and spectral information. This approach has enabled morphological and biological tissue analysis for applications including cancer detection, ophthalmology, and microscopy.^3^^,^^19^^–^^29^ Multispectral imaging utilizes only a few wavelengths (typically < 10),^23^^,^^30^ whereas hyperspectral imaging^20^^,^^24^^–^^29^^,^^31^^–^^34^ provides full spectroscopic sampling (typically > 100 wavelengths). High-resolution hyperspectral imaging thus generates very large datasets (e.g., 1,000,000 spatial pixels × 100 wavelengths), typically requiring dimensionality reduction and machine learning for analysis.^20^^,^^24^^,^^35^

When spectral imaging is based on collecting diffusely reflected light from an object, the spectrum measured at each image pixel depends on the object’s local absorbing and scattering properties. In biological tissue, oxyhemoglobin, deoxyhemoglobin, melanin, water, and other tissue components have optical properties that are tabulated in the literature.^36^^,^^37^ Given a measurement of an object’s diffuse reflectance and knowledge of the absorption and reduced scattering spectra of these pure components, an estimate of the concentration of each component within the measured tissue can be made. This can be done using a variety of fitting algorithms including least squares regression.^38^^–^^40^ However, iterative fitting approaches can often be computationally intensive.^39^^–^^42^ To reduce computation time, machine-learning approaches have been developed for predicting blood volume, oxygen saturation, epidermal thickness, and melanin from hyperspectral images of human skin.^41^^,^^43^ These methods rely on training data generated through analytical or numerical simulations^4^^,^^42^^–^^44^ or on hyperspectral image data acquired from *in vivo* studies. In each of these previous studies, the wavelength-dependent scattering intensity was either assumed or not predicted despite the variation of scattering among tissues and individuals. However, predicting scattering rather than assuming it is crucial as it significantly enhances the accuracy of component predictions by capturing its variability. This approach acknowledges the interplay between scattering and absorption, leading to more reliable and precise estimations of all tissue components. By accurately predicting scattering, the overall model becomes more robust, reflecting the true optical properties of the tissue. Unlike the previously mentioned studies, by incorporating a scattering intensity prediction, this work aims to provide a more comprehensive analysis of tissue properties and extend the analysis beyond commonly studied tissues, such as skin, to include less commonly studied tissues, such as gingiva.

Gingiva, or gum tissue, is particularly significant due to its role in oral health. Gingivitis, the inflammation of the gums, is a common condition that can lead to more severe periodontal diseases if left untreated.^45^ Detecting gingivitis early is crucial as it can prevent the progression to periodontitis, which is associated with tooth loss and other systemic health issues such as cardiovascular disease and diabetes.^46^^,^^47^ Therefore, the ability of our device and model to detect gingivitis can have substantial clinical implications, improving patient outcomes through early intervention and management of oral health.

Here, two separate artificial neural networks (ANNs) were trained on simulated data to predict the blood volume, oxygen saturation, melanin content, and scattering intensity of human skin and gingival tissue from simulated diffuse reflectance spectra. The trained model was then applied to real diffuse reflectance spectra obtained from *in vivo* hyperspectral imaging of tissue. Occlusion was used to induce changes in tissue oxygenation by temporarily removing the arterial supply, abrasion was used to induce inflammation and produce changes in blood content and oxygenation, and subjects with visibly different levels of melanin were imaged to analyze the effects of pigmentation. The method presented here combines high spatial and spectral resolution hyperspectral imaging with neural network analysis to predict *in vivo* blood volume, oxygen saturation, melanin content, and scattering intensity for both skin and gingival tissues. The increasing availability of robust hyperspectral imaging hardware paired with rapid neural network-based analysis may enable new applications in non-invasive tissue assessment in the clinical setting.

## 2 Materials and Methods

### 2.1 Hyperspectral Imaging Setup

**Fig. 1:**
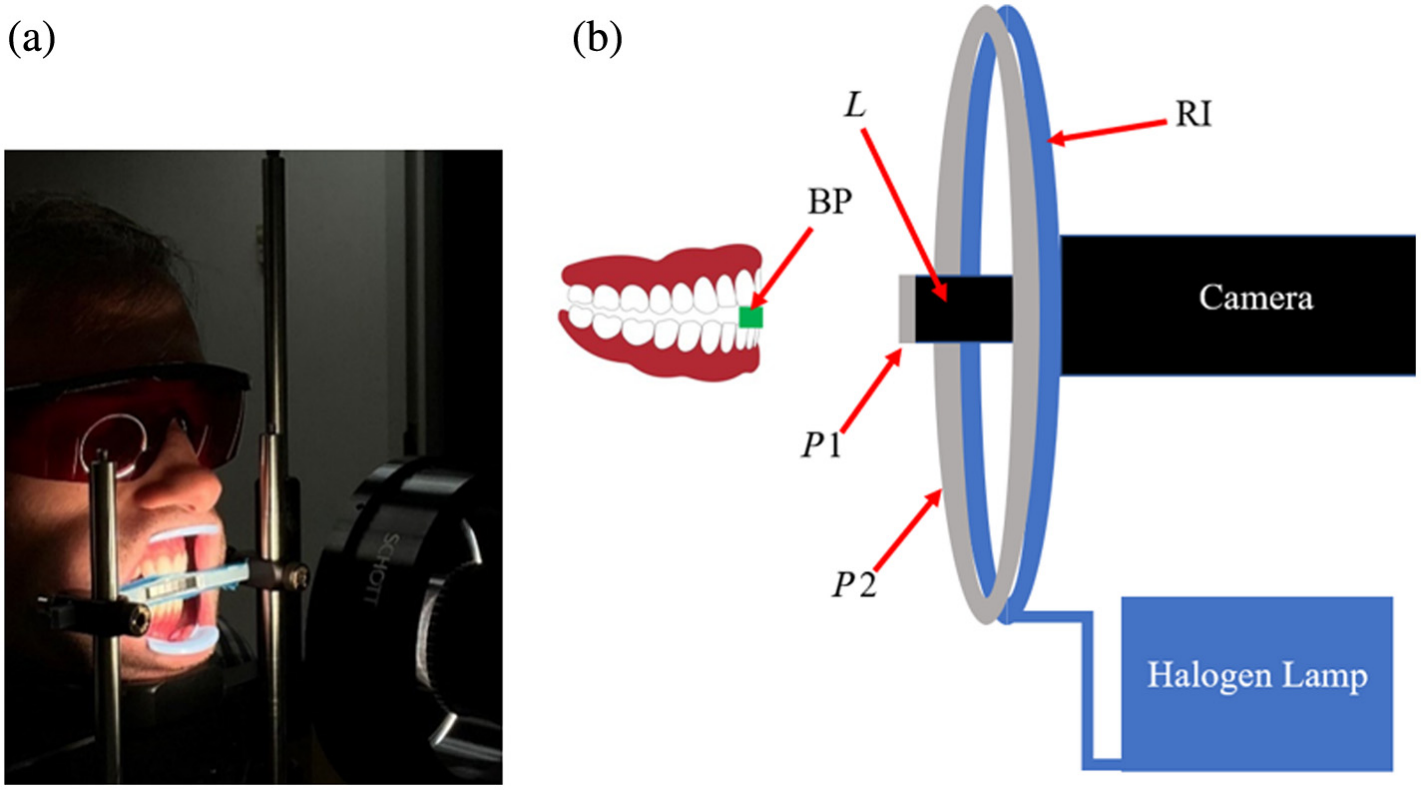
(a) Hyperspectral extraoral imaging system showing subject biting bite piece. (b) Hyperspectral extraoral imaging system hardware diagram. BP is the bite piece for the subject’s teeth with diffuse reflectance standards facing the camera, L is the lens on the camera, RI is the ring illuminator, and P1 and P2 are linear polarizers mounted on the camera lens and ring illuminator, respectively, with their transmission axes orthogonal.

Figure 1 shows the hyperspectral imaging setup used in this study. The camera is a TruTag Technologies Hinalea 4250 (1216×1936  pixels) with a 45 to 50 mm focal length lens (f/5.6) providing a 15 deg field of view. This camera uses a Fabry–Pérot interferometer to collect images at 299 wavelengths from 400 to 1000 nm with a full width at half maximum (FWHM) bandwidth of 4 nm. The light source was a Schott DCR III halogen lamp connected to a 110 mm diameter ring illuminator with a mounted linear polarizing film. An orthogonal linear polarizer (Heliopan, P/N 703030, Gräfelfing, Germany) mounted on the camera lens prevents the acquisition of specular reflection from the sample. The extraoral setup includes a bite piece at a working distance of 150 mm, which serves to stabilize the jaw during image acquisition. On the camera side of the bite piece is a set of six 5×5  mm calibrated diffuse reflectance standards, mounted at the same working distance as the tissue or sample being imaged.

### 2.2 Preprocessing and Calibration

**Fig. 2:**
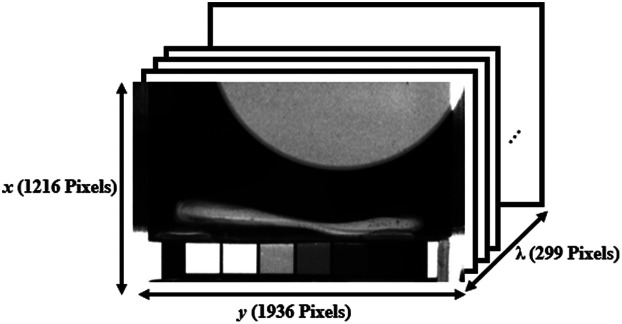
Hyperspectral data cube for a large circular reflectance standard imaged with six square in-frame reflectance standards. The data cube contains 1216×1936 spatial pixels and 299 wavelengths ranging from 400 to 1000 nm.

The camera acquires raw images at 450 different Fabry–Pérot mirror spacings. Each spacing permits light at multiple distinct wavelengths to reach the sensor. The TruScope NRT v1.9.1 software provided by the camera manufacturer is used to process the raw dataset into images at each of 299 wavelength bands in the range 400 to 1000 nm, resulting in a 1216×1936×299 (x,y,λ) data cube (Fig. 2).

Illumination non-uniformity was corrected by first capturing a data cube $I_{\mathrm{std}}$ (x,y,λ) for a uniform 50% diffuse reflectance standard covering the entire field of view. $I_{\mathrm{std},\max}$ (λ) is the maximum pixel value measured at wavelength λ. Raw images of unknown objects, $I_{\text{raw}}$ (x,y,λ), were then corrected for illumination non-uniformity according to Eq. (1), giving a corrected data cube, $I_{c}$ (x,y,λ)

$$I_{c}(x,y,\lambda )=I_{\text{raw}}(x,y,\lambda )\frac{I_{\mathrm{std},\max}(\lambda )}{I_{\mathrm{std}}(x,y,\lambda )}.$$ (1)

Diffuse reflectance standards (FOSS) with manufacturer-specified reflectance values of 0.02, 0.10, 0.20, 0.40, and 0.80 were imaged to determine the wavelength-dependent reflectance of the grayscale tiles mounted on the camera side of the bite piece (Fig. 2). The sum of the pixel values within 40×40  pixel regions were calculated for each of the reflectance standards and each of the grayscale tiles in the field of view. The diffuse reflectance values of the grayscale tiles were then determined based on the values obtained from the reflectance standards.

A second calibration relationship was then developed to convert corrected object pixel values to diffuse reflectance at each wavelength using the bite-piece grayscale tiles that are captured in each tissue image. A data cube was acquired for the grayscale tiles and corrected for illumination non-uniformity according to Eq. (1). At each wavelength, a linear least squares fit was applied to the six data points of measured pixel value versus known diffuse reflectance, providing a pair of calibration parameters (slope and intercept) for each wavelength, m(λ) and b(λ). The diffuse reflectance of subsequent objects, $R_{d}(x,y,\lambda )$, was determined at each wavelength from the corrected pixel value image $I_{c}$ (x,y,λ) according to Eq. (2)

$$R_{d}(x,y,\lambda )=\frac{I_{c}(x,y,\lambda )-b(\lambda )}{m(\lambda )}.$$ (2)

### 2.3 Training and Testing an Artificial Neural Network for Spectral Analysis

**Fig. 3:**
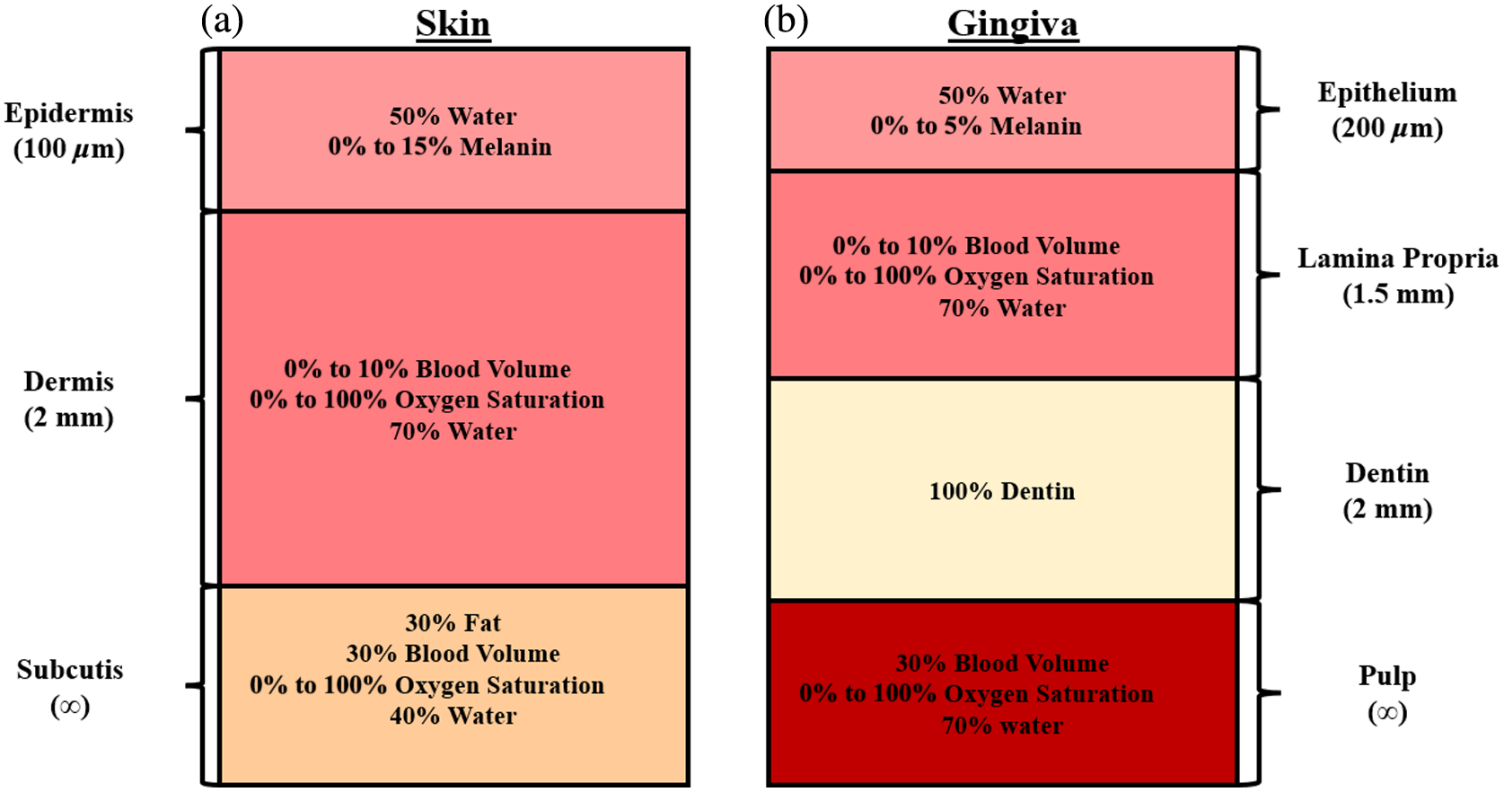
Skin and gingiva models used for Monte Carlo simulations to train the ANN. The skin (a) was modeled with three layers (epidermis, dermis, and subcutis). The gingiva (b) was modeled with four layers (epithelium, lamina propria, dentin, and pulp).^48^^,^^49^

**Fig. 4:**
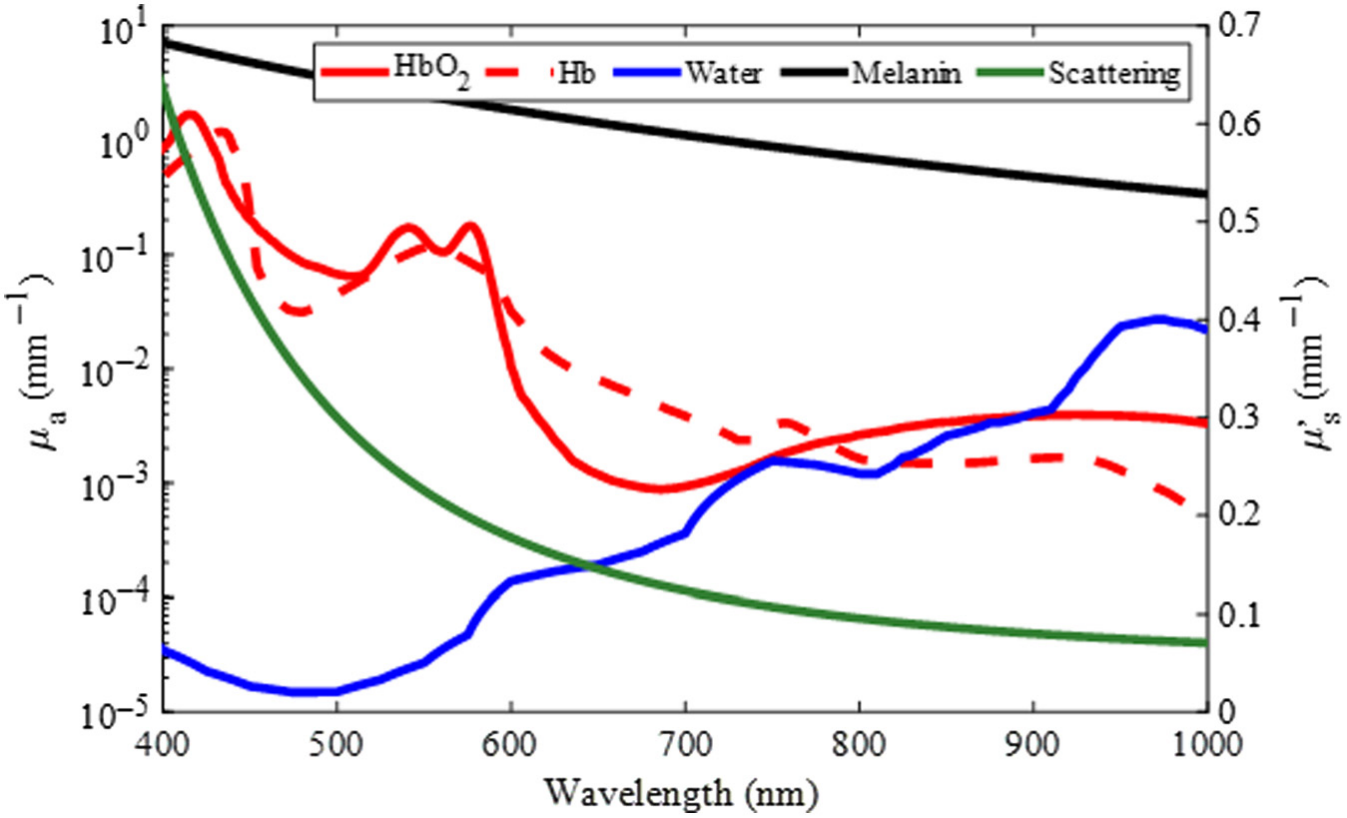
Absorption and reduced scattering coefficients of typical human tissue components given a blood volume of 1%, oxygen saturation of 60%, water content of 60%, melanin content of 5%, and scattering intensity of $0.3\;{\mathrm{mm}}^{-1}$.

Each spatial pixel in a calibrated hyperspectral data cube contains the diffuse reflectance spectrum $R_{d}(\lambda )$ at the object location being imaged. To estimate the blood volume (B), oxygen saturation (S), scattering intensity (a), and melanin content ($f_{m}$) at each pixel, an ANN was developed. To train the ANN, simulated diffuse reflectance spectra were generated using two separate Monte Carlo models: one for skin and one for gingiva (Fig. 3).^50^ First, absorption and reduced scattering coefficients were calculated by selecting a physiologically relevant combination of B, S, $f_{m}$, a, and water content (W)^36^^,^^51^ and combining these with their respective extinction coefficients, according to Eqs. (3) and (4).^52^ Rather than the subcutis layer that is present in the skin, dentin and pulp layers were added to simulate the tooth under the gingiva.^48^^,^^49^^,^^53^ Both the skin and gingiva models included a constant background absorption in the top two layers.^4^^,^^40^^,^^51^ Example spectra are shown in Fig. 4 for the specific choices of B=1%, S=60%, W=60%, $f_{m}=5\%$, and $a=0.3\;{\mathrm{mm}}^{-1}$. The ranges of physiological values were selected to represent a variety of skin pigmentations, skin compositions, and tissue structures.^36^^,^^37^^,^^51^^,^^52^^,^^54^

A tissue’s overall absorption coefficient $\mu_{a}(\lambda )$ is given by

$$\mu_{a}(\lambda )=\overset{n}{\underset{i=1}{\sum }}c_{i}\varepsilon_{i}(\lambda ),$$ (3)

where λ represents wavelength, $c_{i}$ is the concentration, and $\varepsilon_{i}$ is the extinction coefficient of absorber i. Similarly, the tissue’s reduced scattering coefficient $\mu_{s}^{\prime }(\lambda )$ was modeled by

$$\mu_{s}^{\prime }(\lambda )=a(\frac{\lambda }{500\;\mathrm{nm}}{)}^{-b},$$ (4)

where a is the scattering intensity and b=2.^52^

These absorption and reduced scattering coefficients were used as inputs to the multi-layer Monte Carlo model, generating a diffuse reflectance spectrum $R_{d}(\lambda )$ for the specific set of B, S, $f_{m}$, and a values.

**Fig. 5:**
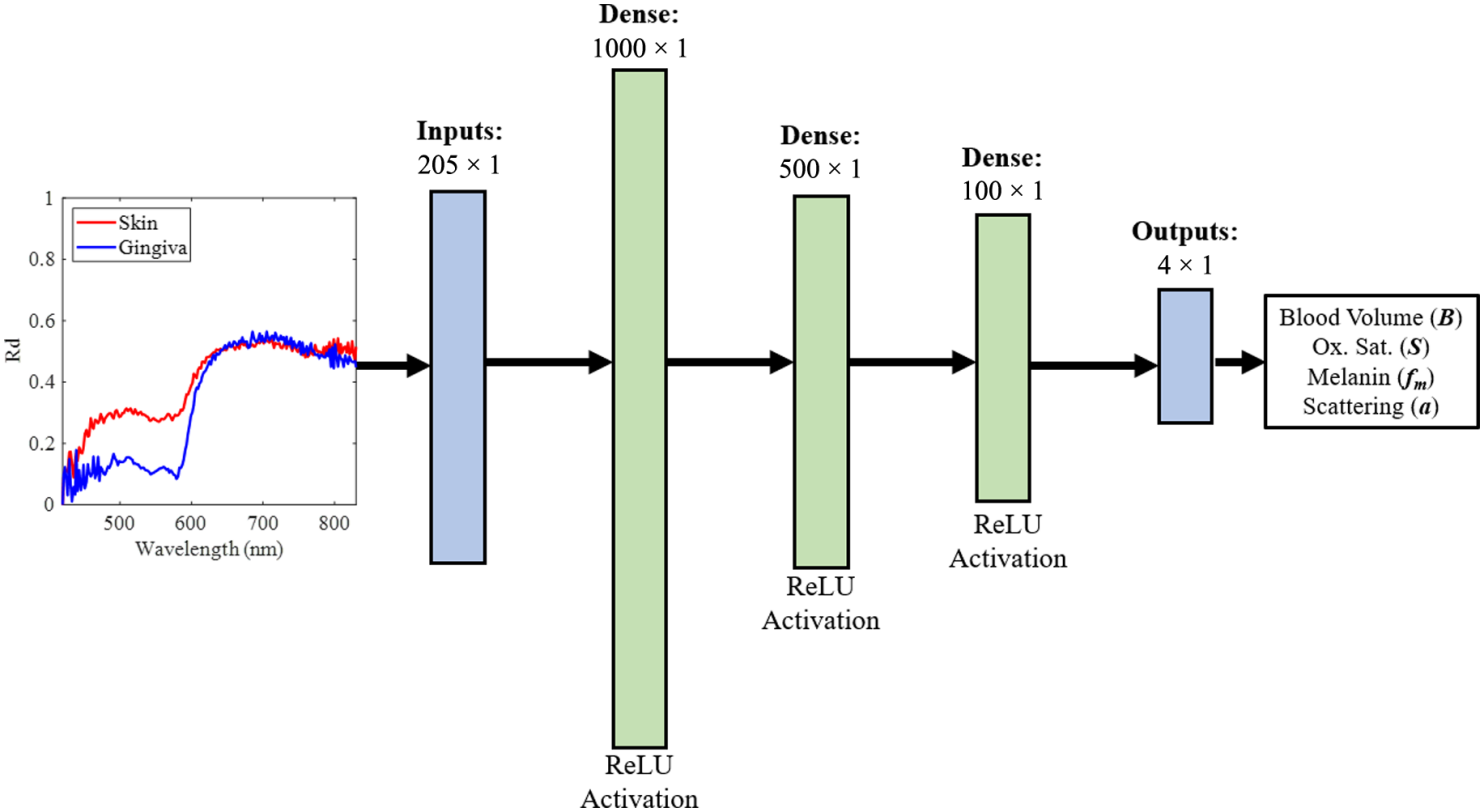
Structure of the ANN. The input is a diffuse reflectance spectrum with values at 205 wavelengths in the range [420, 830 nm]. Example skin (red) and gingiva (blue) reflectance spectra are shown as inputs to the ANN. These spectra were obtained from 2×2 regions of experimental data captured by the system shown in Fig. 1. The three dense middle layers of the ANN each utilize a rectified linear unit (ReLU) activation function. The 4×1 output of the ANN contains the log (blood volume), oxygen saturation, melanin content, and the scattering intensity.

In this work, the tissue refractive index (n) was held constant at 1.4, epidermal water content was held constant at 50%, and dermal water content was held constant at 70%. For the skin model, the epidermal thickness was held constant at 100  μm, the dermal thickness was held constant at 2 mm, and the subcutis was semi-infinite.^8^^,^^37^^,^^52^^,^^55^^,^^56^ In the gingiva model, the epithelial thickness was held constant at 200  μm, lamina propria thickness was held constant at 1.5 mm, dentin was held constant at 2 mm, and the pulp was semi-infinite.^48^^,^^49^ This method was used to generate 30,000 simulated diffuse reflectance spectra, $R_{d}(\lambda )$, for each tissue model. Gaussian noise with varying standard deviations was added to the simulated spectra to replicate experimental conditions, resulting in average signal-to-noise ratios ranging from ∼10 to 16 dB. The 30,000 simulated diffuse reflectance spectra were separated into 19,200 spectra to train the ANN, 4800 spectra to validate the ANN, and 6000 spectra to test the ANN. Due to the output wavelength range from the halogen light source and the optimal collection range of the hyperspectral camera, reflectance spectra were generated in the range of 420 to 830 nm, resulting in a total of 205 wavelengths used as input for the ANN. The diffuse reflectance spectra were scaled at each wavelength across the entire training set using the min–max scaling method provided by the scikit-learn Python library.^57^ The same scaling method was applied to the B, S, $f_{m}$, and a values in the entire training set. Outputs of the ANN were then unscaled to produce the predicted values of the tissue properties. The ANN structure (Fig. 5) consisted of three hidden layers and was tested using the previously mentioned independent set of 6000 simulated diffuse reflectance spectra that were not included in the training step. To determine the size and number of the dense layers in the ANN, the complexity of the input data, output data, and function being fit were all taken into consideration. The size and number of dense layers that were chosen yielded the lowest root-mean-square prediction errors among the networks tested. The rectified linear unit activation (ReLU) was utilized in each hidden layer. The ANN was trained and tested using an AMD Ryzen 7 4700U CPU.

### 2.4 *In Vivo* Experiments

To test the ability of the ANN to predict B, S, $f_{m}$, and a values for tissue *in vivo* and detect changes in these values, experiments on skin and gingiva were performed on three subjects under an Institutional Review Board approved protocol (U.S.IRB2023CP/04). First, to show the ability to measure changes in oxygen saturation, two fingers were imaged in the same field of view at the same plane as the in-frame grayscale calibration bar (Fig. 1). Blood flow in one finger was occluded for 5 mins using a rubber band before being released, whereas the other finger was unimpeded. Next, the ability to detect small differences in tissue oxygen saturation and melanin content was demonstrated by performing a 10-s finger occlusion in subjects with a range of pigmentation levels. To demonstrate the measurement of physiological changes in gingival tissue, inflammation was induced on one lateral side of the mouth by 1 min of brushing with a stiff-bristle toothbrush, whereas the contralateral side remained unbrushed. Finally, a change in blood volume (B) was shown with a corresponding decrease in gingivitis over a 3-week timespan from a clinical study investigating oral health.

## 3 Results

### 3.1 Simulated Experiments

**Fig. 6:**
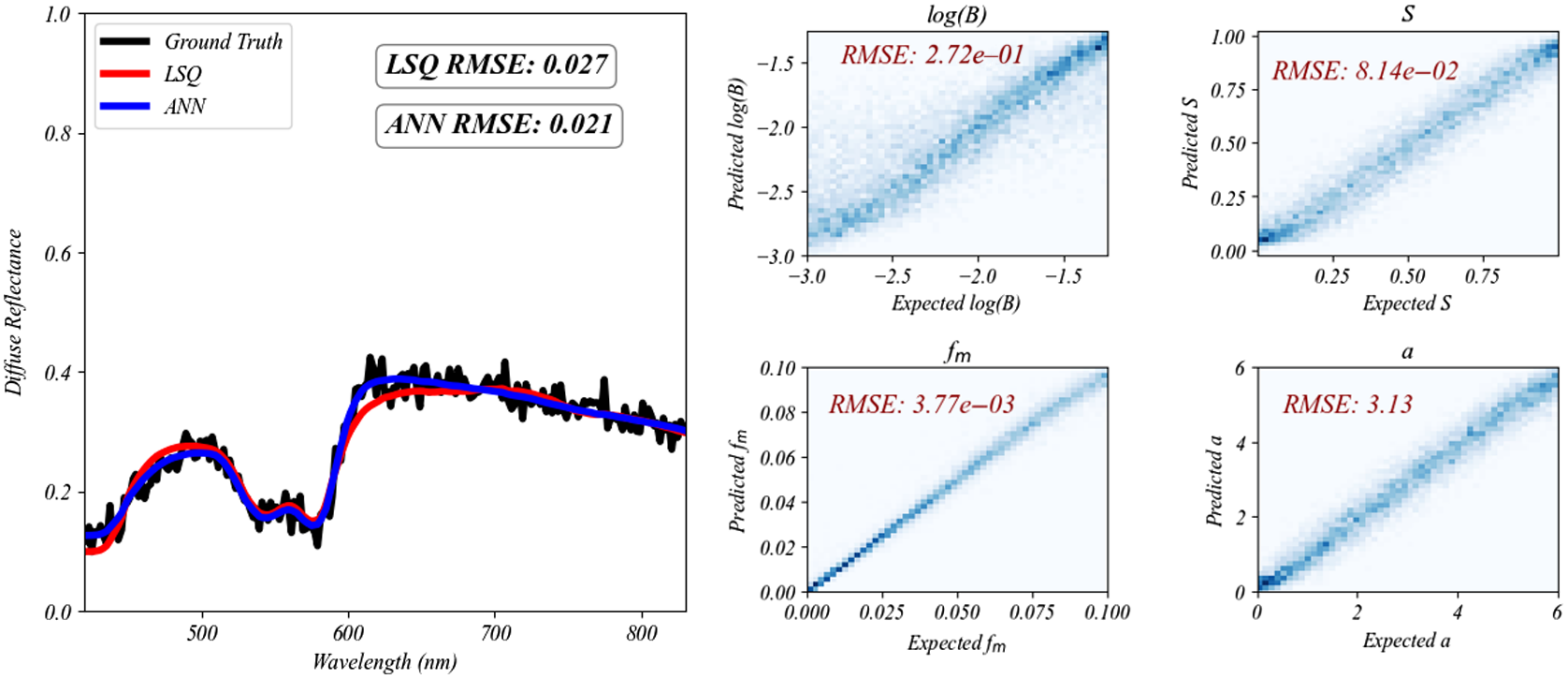
Example test spectrum with ANN and iterative least squares (LSQ) fits are plotted on the left. The ANN fit all 100 test spectra with an average RMSE of 0.021, whereas LSQ fit the spectra with an average RMSE of 0.027. The plots on the right show predicted versus ground truth 2D histograms of B, S, $f_{m}$, and a for 6000 simulated reflectance spectra, obtained from the ANN. The RMSE values for each parameter are shown on their respective plots. The ANN made its predictions for the entire test set of 6000 spectra in 0.4 s.

The ANN was trained in 4 mins and 38 s on an AMD Ryzen 7 4700U CPU and tested using 6000 diffuse reflectance spectra generated from known B, S, $f_{m}$, and a values. Using these reflectance spectra as input, the ANN model predicted B, S, $f_{m}$, and a values with low root mean squared error (RMSE) values (Fig. 6). Furthermore, the ANN was compared with Monte Carlo iterative least squares regression using another separate test set of 100 gingiva Monte Carlo simulations with added noise. The ANN and Monte Carlo iterative least squares regression each performed very well with the fitted spectra averaging RMSE of 0.021 and 0.027, respectively. The average time required for the ANN to analyze one spectrum was only 0.07 s on the AMD Ryzen 7 4700U CPU, whereas the Monte Carlo iterative least squares approach took significantly longer, averaging 301.74 s per spectrum on an NVIDIA GeForce RTX 3080 Ti Laptop GPU, making it over 4000 times slower.

### 3.2 *In Vivo* Experiments

**Fig. 7:**
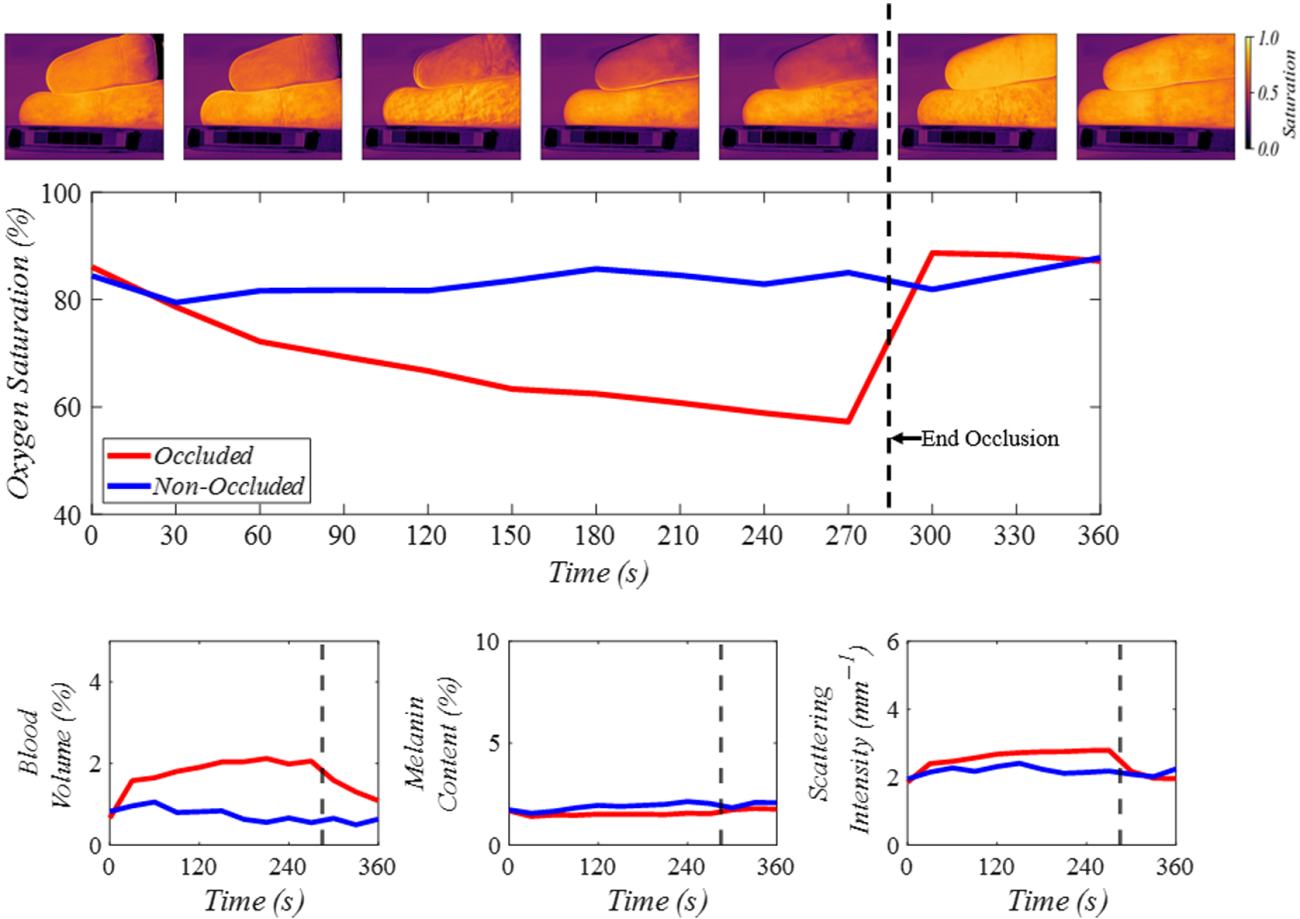
Predicted oxygen saturation (S), blood volume (B), melanin ($f_{m}$), and scattering intensity (a) for 80×80  pixel regions of interest on the occluded and non-occluded fingers. The images along the top of the plot correspond to the oxygen saturation maps from the ANN at every 60 s. The occluded finger is at the top of each image, and the non-occluded finger is at the bottom of each image. Average oxygen saturation values from a region of interest on each finger are shown in the large plot below the oxygen saturation images. Blood volume, melanin content, and scattering intensity are shown in the bottom three plots.

Figure 7 shows the results of the first finger occlusion experiment, where a region of interest was selected on both the occluded and non-occluded fingers. The images shown along the top of the plot show the decrease in oxygen saturation of the occluded finger (top finger) over time, whereas the non-occluded finger (bottom finger) remained the same. This is shown in the plot as well where oxygen saturation of the occluded finger gradually decreases until the rubber band is released at the 285-s time point, at which time, the oxygen saturation spikes and begins to return to the initial state. The blood volume also showed elevated levels in the occluded finger immediately after the occlusion began. The melanin content remained relatively unchanged, as expected. Scattering intensity showed small changes before and after occlusion while remaining consistent with the expected *in vivo* results.^52^

**Fig. 8:**
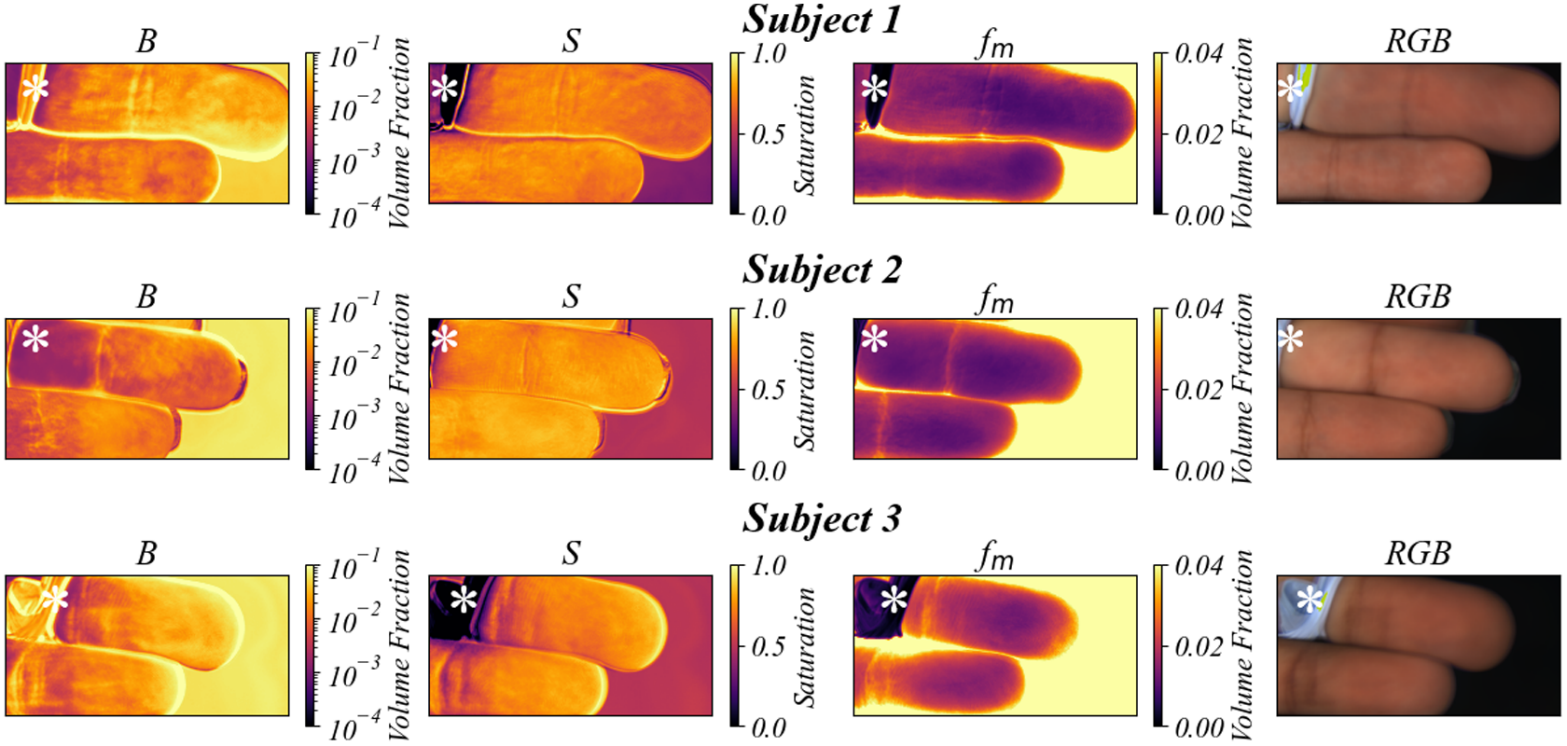
Predicted blood volume (B), oxygen saturation (S), melanin ($f_{m}$), and corresponding RGB images for the pigmentation/occlusion experiment. The top finger was occluded by a rubber band that is indicated by the white asterisk in each image, and the middle finger was not occluded. Each subject’s skin was assigned a score based on the Fitzpatrick skin type scale, where I is least pigmented and VI is most pigmented. Subject 1 showed Fitzpatrick type II, subject 2 showed type III, and subject 3 showed type IV.

Figure 8 shows the blood volume, oxygen saturation, and melanin maps predicted using the ANN for a 10-s finger occlusion of three subjects with visibly different levels of pigmentation. The pigmentation differences are further shown in an additional RGB image for each subject. The occluded finger, shown at the top of the images, shows slightly lower oxygen saturation than the non-occluded finger for each subject, as expected. In addition, higher melanin content was measured in the subjects with darker skin pigmentation.

By selecting a region of interest on each fingertip, the average blood volume, oxygen saturation, and melanin content were calculated for each subject and can be seen in Table 1. The blood volume, melanin content, and scattering intensity were relatively unchanged between the occluded and non-occluded fingers for each subject. However, the oxygen saturation level in the occluded finger was lower than the non-occluded finger for all subjects. Given that the occlusion only lasted 10 s, the oxygen saturation only decreased by ∼3% to 5% for each subject. The measured melanin content for subject 1 was the lowest followed by subjects 2 and 3, as expected.

**Table 1:** Average tissue component values for regions of interest on occluded and non-occluded fingers.

Subject	Tissue component	Non-occluded finger	Occluded finger
1	Blood volume, B	1.23%	2.83%
	Oxygen saturation, S	73.95%	69.22%
	Melanin, $f_{m}$	1.63%	1.70%
	Scattering intensity, a	$2.27\;{\mathrm{mm}}^{-1}$	$2.71\;{\mathrm{mm}}^{-1}$
2	Blood volume, B	1.26%	0.74%
	Oxygen saturation, S	82.37%	78.86%
	Melanin, $f_{m}$	2.58%	2.43%
	Scattering intensity, a	$2.54\;{\mathrm{mm}}^{-1}$	$2.24\;{\mathrm{mm}}^{-1}$
3	Blood volume, B	2.86%	3.44%
	Oxygen saturation, S	80.03%	77.98%
	Melanin, $f_{m}$	2.88%	3.14%
	Scattering intensity, a	$3.45\;{\mathrm{mm}}^{-1}$	$3.41\;{\mathrm{mm}}^{-1}$

**Fig. 9:**
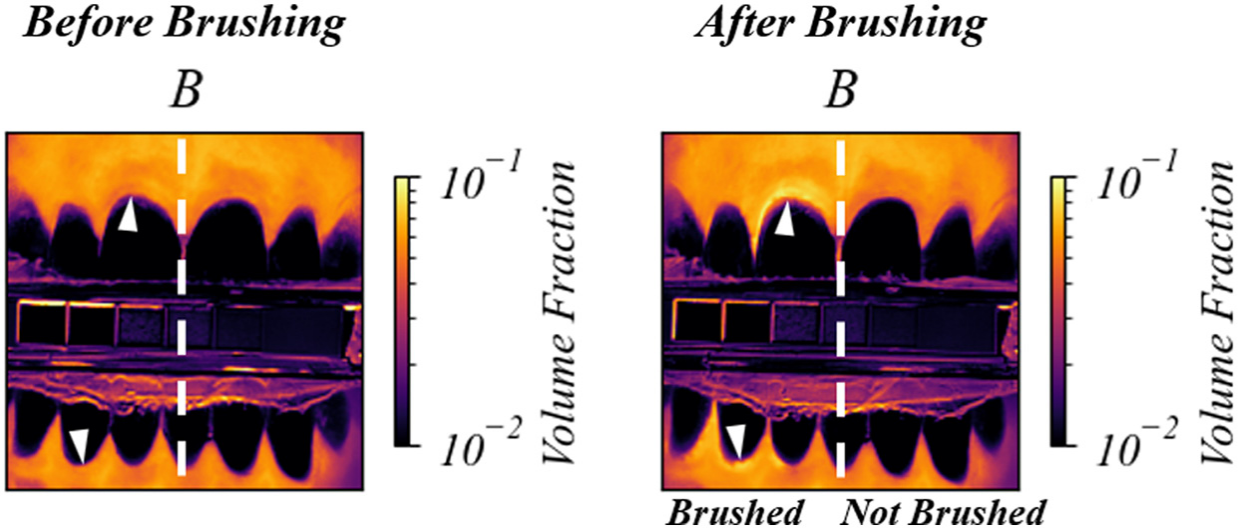
Predicted blood volume (B) for a human subject pre- and post-brushing. The teeth and gingiva on the left side of the mouth were brushed with a stiff-bristled toothbrush for 1 min. The increase in blood volume after brushing is indicated by arrowheads on the left side of the white-dashed line in the gingiva images.

The effects of abrasion on gingival tissue are apparent in Fig. 9. Teeth and gingiva only on the left side of the images were brushed. The white arrowheads indicate regions where the blood content (B) is higher on the brushed (left) side of the gingiva than the not brushed (right) side.

**Fig. 10:**
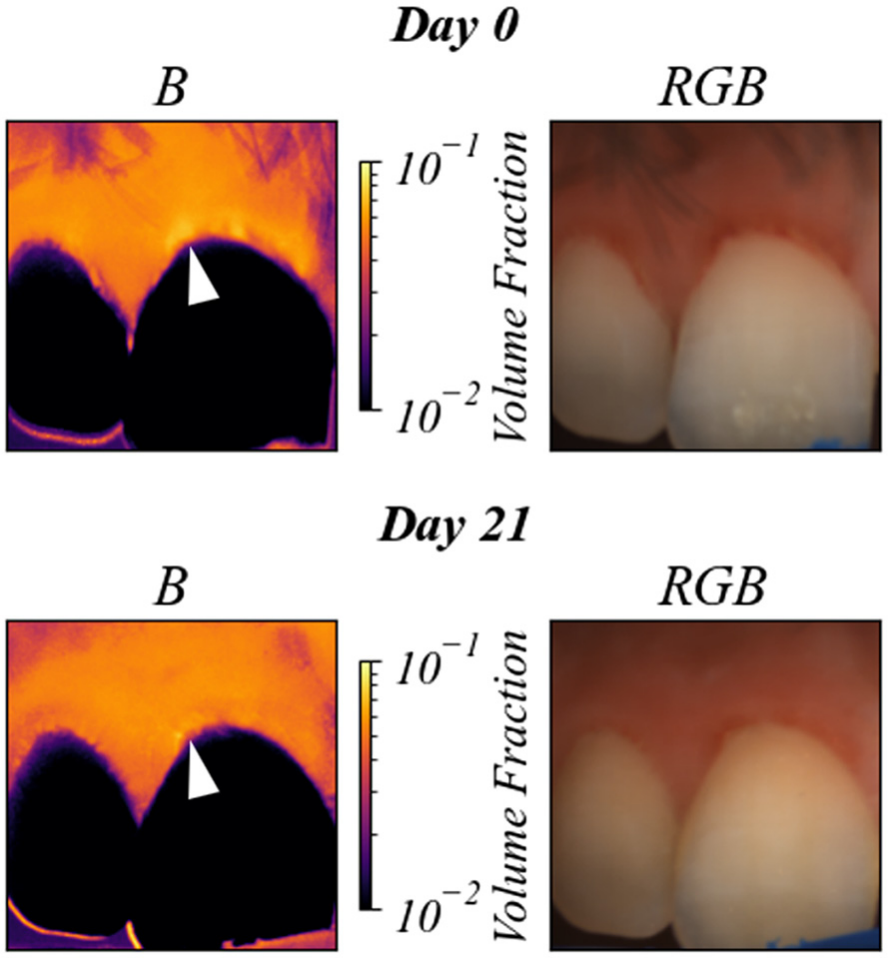
Predicted blood volume (B) for a human subject with gingivitis, before and after 3 weeks of improved oral health. The white arrow indicates the region of decreased blood volume. Changes in B on the surface of the tooth should be disregarded due to the fact that the ANN was not trained on teeth spectra.

The increase in blood volume shown in Fig. 9 is an effect of toothbrush abrasion causing inflammation in the gingival tissue. Figure 10 shows the gingival tissue above a tooth afflicted with gingivitis before and after a 3-week regimen of improved oral health. The images show a clear decrease in blood volume, consistent with a decrease in inflammation.

## 4 Discussion

A hyperspectral imaging system was assembled to capture images at 299 wavelengths between 400 and 1000 nm. The acquisition time for the whole data cube was 25 s, with individual image exposure times of 40 ms, plus a short interval due to tuning of the Fabry–Pérot interferometer. Subject movement during image acquisition was found to be negligible due to the stabilization bar.

Illumination uniformity correction and calibration using in-frame reflectance standards ensured accurate calculation of the pixel-by-pixel reflectance spectra. The multi-layer Monte Carlo approach relied on accurate calibration for the detection of changes in the optical properties and tissue components. The unique spectra of the main absorbers in the visible wavelength range (oxyhemoglobin, deoxyhemoglobin, and melanin) were key factors in successful spectral fitting. This was clear in the finger occlusion shown in Fig. 7 where the melanin was relatively unchanged between the occluded and non-occluded fingers, but the oxygen saturation map showed clear differences. By incorporating melanin in the spectral analysis, this system and ANN analysis are suited for imaging skin with a range of pigmentations for tissue component estimation, as demonstrated in Fig. 8. These experiments demonstrated the potential of this system for use in both human skin and gingival tissue *in vivo*. The finger occlusion tests not only validated the system’s ability to differentiate tissue parameters effectively but also demonstrated the importance of including scattering intensity in the spectral fitting. The scattering intensity showed consistency with expected *in vivo* measurements, reinforcing the system’s robustness. However, the lack of experimental confirmation that predicting scattering improves model robustness suggests that further validation with *in vivo* data is necessary. Although this system successfully demonstrated the ability to monitor these parameters *in vivo*, these results cannot be used to gauge the accuracy of this system due to the lack of an *in vivo* ground truth. Additional subjects will be necessary to fully assess the clinical relevance and effectiveness of this device.

Despite the camera’s ability to collect wavelengths from 400 to 1000 nm, the current ANN used wavelengths from 420 to 830 nm due to the low signal-to-noise ratio obtained at the shorter and longer wavelengths. A different light source that covers the full 400 to 1000 nm range can possibly improve the ANN predictions by incorporating the short wavelengths where blood and melanin absorption are higher while also opening the possibility of including water content in the ANN prediction with longer wavelengths. In addition, other models can be explored, such as random forests, in the future to potentially enhance prediction accuracy, robustness, and speed. The multi-layer Monte Carlo approach for generating ANN training data can also be improved by generating data from multilayered Monte Carlo simulations that include more layers. By doing so, the ANN may be further tailored to tissues with different physiological behaviors. In addition, incorporating training data to model specific conditions and diseases can help capitalize on this method as a fast diagnostic technique.

Although neural networks are commonly used for tissue parameter quantification, the specific focus on skin and gingival tissues and the integration of a multi-layer Monte Carlo model with hyperspectral imaging present a novel contribution. This comprehensive approach enhances the accuracy and applicability of the system, providing a robust framework for tissue parameter estimation. Future work will focus on obtaining ground truth values for *in vivo* data to provide more rigorous validation and developing a more generalized model capable of accurately quantifying tissue parameters for both skin and gingiva.

## 5 Conclusion

This study reported a unique and versatile method for the prediction of blood volume, oxygen saturation, melanin content, and scattering intensity in both skin and gingival tissue. The hyperspectral camera provides high spatial and spectral resolution. The ANN provides high-speed analysis that can be used in the clinic non-invasively on a variety of human tissues *in vivo*. Future work aimed at enhancing validation and expanding the model’s applicability to multiple tissue types will further establish the clinical relevance and effectiveness of this imaging system.

## Data Availability

All relevant code, data, and materials are available from the authors upon reasonable request. Correspondence and requests should be addressed to the corresponding author.
